# Dissecting the Spectrum of Stroke Risk Factors in an Apparently Healthy Population: Paving the Roadmap to Primary Stroke Prevention

**DOI:** 10.3390/jcdd10020035

**Published:** 2023-01-20

**Authors:** Daniela Efremova, Dumitru Ciolac, Eremei Zota, Danu Glavan, Natalia Ciobanu, Wolfgang Aulitzky, Anna Maria Nics, Eugen Trinka, Chiaki Yamada, Alexandru Movila, Stanislav A. Groppa

**Affiliations:** 1 Department of Neurology, Institute of Emergency Medicine, 2004 Chisinau, Moldova; 2 Department of Neurology, Nicolae Testemitanu State University of Medicine and Pharmacy, 2004 Chisinau, Moldova; 3 Open Medical Institute, American Austrian Foundation, 5020 Salzburg, Austria; 4 Department of Neurology, Christian Doppler University Hospital, Paracelsus Medical University, Centre for Cognitive Neuroscience, 5020 Salzburg, Austria; 5 Neuroscience Institute, Christian Doppler University Hospital, Paracelsus Medical University, Centre for Cognitive Neuroscience, 5020 Salzburg, Austria; 6 Department of Public Health, Health Services Research and Health Technology Assessment, UMIT—University for Health Sciences, Medical Informatics and Technology, 6060 Hall in Tirol, Austria; 7 Department of Biomedical Sciences and Comprehensive Care, Indiana University School of Dentistry, Indianapolis, IN 46202, USA; 8 Indiana Center for Musculoskeletal Health, Indiana University School of Medicine, Indianapolis, IN 46202, USA

**Keywords:** cerebrovascular disease, risk factors, epidemiology, prevention

## Abstract

We aimed to investigate, for the first time, the spectrum of stroke risk factors specific to the population of the Republic of Moldova. The subjects were examined according to a pre-established protocol of risk factor estimation. The study involved 300 subjects, including 60% women and 40% men, with a mean age of 49.9 ± 14.5 years. The most common risk factor was abdominal obesity, identified in 75% of subjects; general obesity was detected in 48%, while 32% of subjects were overweight and 20% were normally weighted. Hypertension was observed in 44%; 8% of those examined had atrial fibrillation, and 9% had diabetes mellitus. Left myocardial hypertrophy on ECG was present in 53% of subjects, and acute ischemic changes in 2%. Laboratory observations detected that glycosylated hemoglobin increased by 7%, and >50% had dyslipidemia. Total cholesterol was significantly elevated by 58%, LDL-cholesterol was increased by 32%, and HDL-cholesterol was decreased by 9%. Homocysteine was increased in 55% and high-sensitivity C-reactive protein in 28% of subjects. These results indicate the presence of modifiable risk factors and the necessity to elaborate on the primary prevention strategies aimed at minimizing the burden of stroke in the population of the Republic of Moldova.

## 1. Introduction

Stroke is the second most common cause of death worldwide and a frequent cause of adult disability in developed countries [1,2,3,4]. Stroke has a significant physical, psychological, and financial impact on patients, their families, healthcare systems, and society [5,6,7]. The incidence of stroke in Europe in early 2000 ranged from 95 to 290/100,000 per year [8]. Nearly 75% of stroke cases occur in people aged >65 years [9,10], and about one-third of patients die in the first year following a stroke [11,12]. More than half of stroke survivors remain dependent on caregivers to carry out their everyday activities [13]. Many medical, economic, and social gaps exist, especially in developing countries, that hinder adoption and implementation of effective population-wide stroke preventive programs to reduce the burden of stroke.

Stroke prevention can be classified into primordial, primary, and secondary levels [14]. Strategies to prevent a first stroke address primordial and primary levels, while strategies to prevent recurrent stroke apply to the secondary level. Identifying individuals at risk for stroke requires a detailed definition and evaluation of risk factors for stroke [15,16,17]. Risk factors are categorized into non-modifiable and modifiable risk factors. Whereas non-modifiable risk factors are immutable characteristics inherent in a particular individual, modifiable risk factors can be controlled or eliminated through appropriate lifestyle modifications or targeted medical interventions. Age, sex, race/ethnicity, and family history of stroke are non-modifiable risk factors. Modifiable risk factors are hypertension, atrial fibrillation, diabetes, obesity, hypercholesterolemia, cigarette smoking, alcohol consumption, valvular heart disease, asymptomatic carotid stenosis, physical inactivity, etc. Recent data suggest that increased levels of homocysteine [18] and uric acid [19] may also associated with a higher risk of stroke. Stroke risk factors are age- [20] and sex-specific [21]. Previously, it has been postulated that the spectrum of stroke risk factors differs between young adults (aged 18–49 years), in which rare causes predominate, and older adults (aged >50 years), in which traditional vascular risk factors predominate [22]. However, a growing body of evidence suggests that the increasing incidence of stroke in young adults is associated with the rising prevalence of traditional vascular risk factors such as hypertension, dyslipidemia, diabetes mellitus, and obesity [23]. Therefore, reducing the burden of stroke in the population requires the identification of modifiable risk factors in different age groups and demonstration of the efficacy of risk reduction efforts. Although the prevalence data on stroke risk factors are well established in developed countries, there is a lack of reliable data on risk factors in developing countries.

According to the National Center for Health Management of the Republic of Moldova, during the 2000–2016 years, there was a progressive increase in the incidence and prevalence of cerebrovascular diseases reported per 10,000 population—the incidence increased from 20.4 in 2000 to 26.1 in 2016, and the prevalence from 67.0 to 207.8 during the same period. In the Republic of Moldova, the death rate following a stroke remains one of the highest among European countries, with a death rate of 159.2 per 100.000 persons in 2016 [24]. It is important to mention that stroke is the most preventable neurological disease, and up to 80% of all stroke cases could potentially be reduced with appropriate public health measures [25,26]. Thus, there is a pressing need to estimate the prevalence of risk factors associated with stroke in the Moldovan population and to elaborate targeted and efficient preventive strategies.

Developing strategies for stroke prevention requires the characterization of risk factors in healthy individuals and those with preclinical disease in a structured, systematic, and integrated manner. Determining the spectrum of stroke risk factors specific to the Moldovan population is essential for providing guidance in clinical practice, public health policies, and prioritization of research efforts on a national scale. Therefore, the primary aim of this study was to determine the prevalence of risk factors for stroke, as well as their interrelations and distribution across ages, in an apparently healthy population of the Republic of Moldova.

## 2. Material and Methods

### 2.1. Subjects and Study Design

In the Republic of Moldova, the National State Program on Stroke Risk Factors was launched in 2015 with the commitment to identify the stroke risk factors for primary prevention. The study was conducted on a Moldovan population residing in the central region of the country. The screening program was implemented in two stages. In the first stage, local general practitioners prepared the lists of subjects in their circumscription, and all members aged ≥18 years were recruited for the participation. One or two days before the actual examination, subjects were informed again in a door-to-door manner. In the second stage, subjects were face-to-face interviewed, evaluated according to a predetermined protocol of the risk factors’ estimation, and clinically and preclinically examined.

All clinical and instrumental evaluations were performed by a multidisciplinary team consisting of trained neurologists, internists, rheumatologists, and specialists in functional diagnostics. A total of 337 subjects aged ≥18 years were screened, but only 300 subjects were included into the study, as 37 did not present for laboratory tests. Demographic data, previous medical history, family history of cardiovascular diseases, and behavioral factors were collected using a predesigned questionnaire. Physical examination, laboratory tests, ECG, and Doppler-Duplex ultrasound of the extracranial segment of the carotid artery were also performed. The study was approved by the Ethics Research Committee of the Nicolae Testemitanu State University of Medicine and Pharmacy from the Republic of Moldova (notification no. 70 from 30 June 2020). All subjects provided written informed consent prior to recruitment into the study.

### 2.2. Survey for the Risk Factors

The survey was conducted through face-to-face interviews by trained research team members to collect personal data (age, sex, educational level [27]); behavioral factors (diet [28], physical activity [29], cigarette smoking [30], alcohol consumption [31], explicit drug use [32]); family history of stroke [27] or myocardial infarction; and medical history of hypertension [33], diabetes mellitus [33], stroke, coronary heart disease, and valvular heart disease [34].

### 2.3. Physical Examinations

Body weight and height for body mass index (BMI), waist circumference, blood pressure (BP), and heart rate were measured. Blood pressure was assessed in both upper limbs in the supine and standing positions.

### 2.4. Laboratory Tests

Measurements included serum fasting blood glucose (FBG), glycosylated hemoglobin, total cholesterol (TC), triglycerides (TG), high-density lipoprotein cholesterol (HDL-C), low-density lipoprotein cholesterol (LDL-C), high-sensitivity C-reactive protein (hs-CRP), homocysteine, coagulation state, biochemistry, and complete blood count. All blood samples were sent to the International laboratory Synevo for the analyses.

### 2.5. Ultrasound Measurements

The extracranial segment of the carotid arteries on both sides (common carotid artery—CCA, bifurcation, internal carotid artery—ICA, and external carotid arteries—ECA) were screened by Doppler-Duplex ultrasound to determine the intima–media thickness (IMT), total plaque area, and the degree of stenosis [35]. Measurements of IMT were performed 2 cm proximal to dilatation of the carotid bulb.

### 2.6. Definitions

Hypertension was defined as a self-reported history of hypertension, taking blood pressure-lowering medication, or an average of two resting systolic blood pressure (BP) readings of ≥140 mm Hg and/or diastolic BP ≥90 mm Hg in the field survey [36]. Abdominal obesity was defined clinically as an abdominal circumference of ≥80 cm in women and ≥94 cm in men, as described elsewhere [37]. Obesity was defined as a body mass index (BMI) ≥30 kg/m^2^, overweight as BMI of 25.0–29.9 kg/m^2^, and normal weight as BMI of 18.5–24.9 kg/m^2^ [37]. Atrial fibrillation was defined as reported by the subject or diagnosed by ECG in the field survey [38]. Diabetes mellitus was defined as the use of hypoglycemic medications or a self-reported history of diabetes [39]. Drinking status was classified into three categories according to the subjects’ self-report: no consumption, moderate consumption, and excessive consumption according to the National Institute on Alcohol Abuse and Alcoholism (NIAAA) guidelines [40]. Abnormal levels of the examined biochemical parameters were based on the international lab reference ranges. High glycosylated hemoglobin was defined as values over 6.5 mmol/L. High TC was defined as ≥5.0 mmol/L, high LDL-C as ≥4.16 mmol/L, and low HDL-C as ≤1.04 mmol/L. High homocysteine levels were defined as ≥12 μmol/L and high hs-CRP was considered as >3 mg/L. The IMT values of ≥0.9 mm were considered abnormal. Atherosclerotic plaque on Doppler-Duplex ultrasound was defined as a focal structure that either penetrates into the arterial lumen with 0.5 mm or 50% of the adjacent intima–media complex or has a thickness ≥1.5 mm.

### 2.7. Data Analysis

The prevalence of stroke risk factors was estimated as well as the limits of the prevalence error and of central tendency indicators, and these are presented as numeric values of the lower and upper limits of 95% confidence interval (CI). Normal distribution of the analyzed data was checked by the Kolmogorov–Smirnov test. The differences between the variables were assessed by using the Student’s *t*-test for parametric variables, the Mann–Whitney U test for non-parametric variables, and the chi-square test for categorical variables. The Pearson coefficient was used to assess the relationship between the risk factors. A *p*-value of less than 0.05 was set as statistically significant. Given the reported age-dependent variations in the profile of stroke risk factors [22,23], we were also particularly interested in the prevalence of risk factors in different age groups. Hence, for subsequent subanalyses, subjects were divided into two age groups: (1) 18–49 years (young adults) and (2) >50 years (older adults).

## 3. Results

**Figure 1:**
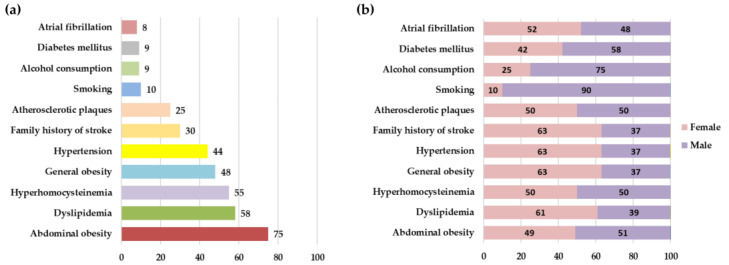
Prevalence of risk factors (%) in the entire study population (**a**) and depending on subject’s sex (**b**).

**Figure 2:**
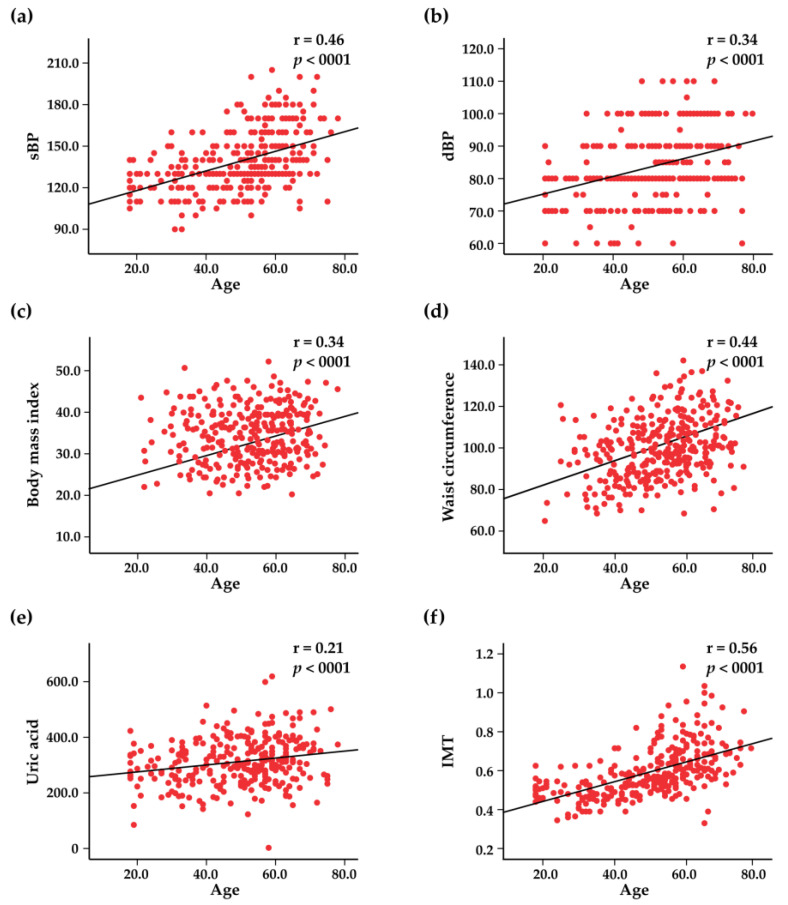
Scatter plots representing the correlations between age and systolic blood pressure (sBP) (**a**), diastolic blood pressure (dBP) (**b**), body mass index (**c**), waist circumference (**d**), blood uric acid levels (**e**), and intima–media thickness (IMT) (**f**).

The study involved 300 subjects aged ≥18 years, including 180 (60%) women and 120 (40%) men, with a mean age of 49.9 ± 14.5 years. There were 122 (41%) subjects in the 18–49 age group and 178 (59%) subjects in the >50 years age group. Characteristics of the study population and prevalence of risk factors are presented separately for men and women in Table 1 and Figure 1. The most frequent risk factors identified in the study population were abdominal obesity (75%), dyslipidemia (58%), hyperhomocysteinemia (55%), general obesity (48%), and hypertension (44%) (Figure 1). Subjects’ age significantly correlated with the following risk factors: systolic BP (r = 0.46, *p* < 0.001), diastolic BP (r = 0.34, *p* < 0.001), BMI (r = 0.34, *p* < 0.001), waist circumference (r = 0.44, *p* < 0.001), mean IMT (r = 0.56, *p* < 0.001), and blood uric acid levels (r = 0.21, *p* < 0.001) (Figure 2).

### 3.1. Smoking

In our study, subjects were asked about their current smoking status, previous experience with smoking, and the amount of daily smoked cigarettes. The overall proportion of current smokers (daily smokers and non-daily smokers) constituted 31 (10%) subjects of all the examined subjects. The proportion of smoking men was much higher compared to that of female smokers (90% vs. 10%, *p* < 0.001). The age group with the highest prevalence of daily smokers and the highest amount of daily smoked cigarettes (55% from total number of smokers) was observed in the 18–49 years age group (Table 2). However, the vast majority of subjects (*n* = 230, 77%) were non-smoking, and 39 (13%) subjects were former smokers.

### 3.2. Physical Activity

Physical activity among the study population was analyzed by estimating the type, duration, and frequency of physical activity per week. In most of the subjects, physical activity presumed household work, with the highest proportion of physical activity being identified in the age group of 18–49 years compared to the >50 years age group (87% vs. 51%, *p* < 0.05).

### 3.3. Arterial Hypertension

One hundred thirty-one (44%) subjects had a positive history of arterial hypertension. The age group most affected by arterial hypertension was >50 years (Table 2), of which 84 (64%) subjects were taking regular antihypertensive treatment, whereas 21 (16%) were not taking any medication and 26 (20%) administered it irregularly. The most compliant to the antihypertensive treatment were women. Mean systolic BP was 139.1 (95% CI: 136.7–141.6)–137.2 mmHg (95% CI: 136.7–141.6) in women and 142.0 mmHg (95% CI: 132.2–145.9) in men. Mean diastolic BP was 83.9 mmHg (95% CI: 82.6–85.2)–82.7 mmHg (95% CI: 82.6–85.2) in women and 85.7 mmHg (95% CI: 83.7–87.7) in men. Mean systolic BP in subjects with arterial hypertension was 153.9 (95% CI: 150.5–157.4) and mean diastolic BP was 90.3 (95% CI: 88.5–92.1).

### 3.4. Atrial Fibrillation

Atrial fibrillation (AF) was identified in 25 (8%) subjects, 15 (60%) of which administered aspirin on a regular basis; 6 (24%) subjects did not receive any medication, and 4 (16%) subjects administered it in an irregular manner. The proportion of women who reported regular aspirin use for the prevention or treatment of cardiovascular diseases was higher compared to men from the same group (73% vs. 27%, *p* < 0.05). Atrial fibrillation was more frequent in the >50 age group (Table 2).

### 3.5. History of Diabetes

A positive history of diabetes was identified in 26 (9%) subjects, the most affected age being >50 years (*n* = 24, 13%). Among the diabetic subjects, 16 (62%) subjects received regular antidiabetic treatment, while 10 (38%) received it in an unsystematic manner. As atrial fibrillation, diabetes was more frequent in the >50 age group (Table 2).

### 3.6. Anthropometric Measurements

**Figure 3:**
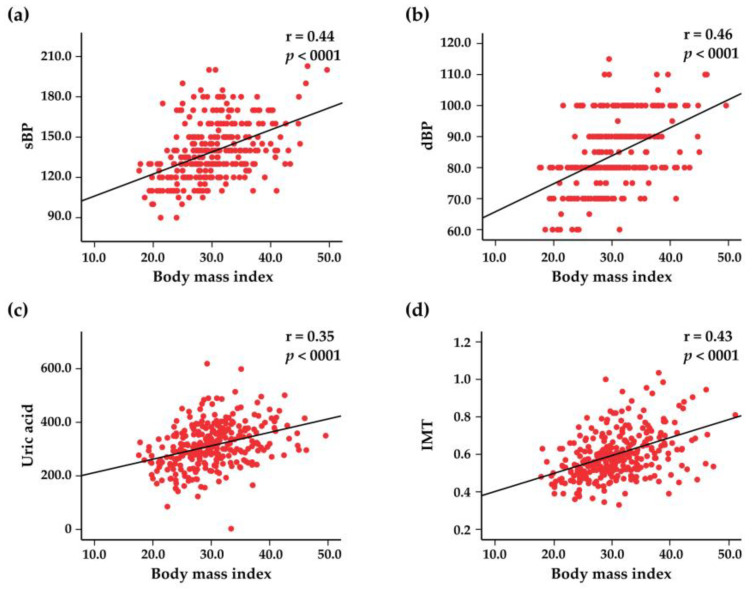
Scatter plots representing the correlations between the body mass index and systolic blood pressure (sBP) (**a**), diastolic blood pressure (dBP) (**b**), blood uric acid levels (**c**), and intima–media thickness (IMT) (**d**).

**Figure 4:**
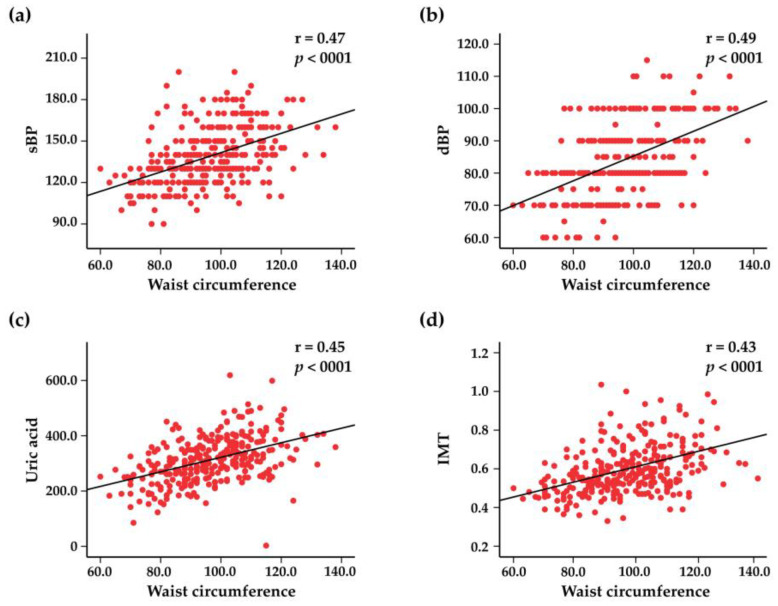
Scatter plots representing the correlations between waist circumference and systolic blood pressure (sBP) (**a**), diastolic blood pressure (dBP) (**b**), blood uric acid levels (**c**), and intima–media thickness (IMT) (**d**).

The mean BMI among the study population subjects of both sexes was 30.1 (95% CI: 29.4–30.7)–29.2 (95% CI: 28.2–30.1) in men and 30.7 (95% CI: 29.8–31.6) in women. Obesity was one of the most common risk factors, being detected in 145 (48%) subjects; 110 (32%) subjects were overweight, and only 60 (20%) had a normal weight. In Figure 3, the associations between BMI and systolic BP, diastolic BP, uric acid, and IMT are represented. Abdominal obesity was present in 75% of subjects, with a mean waist circumference in both sexes of 96.5 (95% CI: 94.8–98.1)—for women, 95.1 (95% CI: 92.7–97.7) and for men, 99.1 (95% CI: 96.6–101.5). Waist circumference positively correlated with systolic BP, diastolic BP, blood levels of uric acid, and IMT (Figure 4).

### 3.7. Glycosylated Hemoglobin and Glucose Levels

The average FBG was 5.4 mmol/L (95% CI: 5.2–5.5), including those currently on antidiabetic medication—5.5 mmol/L (95% CI: 5.3–5.8) in men and 5.3 mmol/L (95% CI: 5.1–5.4) in women. Mean HbA1c values were 5.7 (95% CI: 5.6–5.8)–5.6 (95% CI: 5.5–5.7) in women and 5.7 (95% CI: 5.5–5.9) in men. Elevated HbA1c levels were identified in 7% of the study population, among them, 36% women and 64% men.

### 3.8. Levels of TC, HDL-C, LDL-C, and TG

The mean level of TC in the study population was 5.4 mmol/L (95% CI: 5.3–5.5), of HDL-C—1.4 mmol/L (95% CI: 1.4–1.5), and of LDL-C—3.8 mmol/L (95% CI: 3.6–3.9). Total cholesterol was increased in 58% and LDL-C in 32% of subjects. The HDL-C was decreased in 9% of subjects.

### 3.9. Levels of Hs-CRP and Homocysteine

The mean level of hs-CRP in the study population was 4.0 mmol/L (95% CI: 1.9–7.8), and the mean level of homocysteine was 14.8 mmol/L (95% CI: 13.4–15.9). The hs-CRP levels were elevated in 28% of subjects, and high levels of homocysteine in 55% of subjects were observed.

### 3.10. ECG Results

All the included subjects were examined through ECG, which revealed the following alterations: left ventricular hypertrophy in 159 (53%) subjects, signs of old myocardial infarction in 3%, and acute ischemia in 2%. Atrial fibrillation was identified in 4% of subjects (Table S1). Normal sinus rhythm was present in 95% of subjects (65% women and 35% men), whereas sinus bradycardia constituted 2% and sinus tachycardia 7%.

### 3.11. Doppler Sonography of the Carotid Arteries

**Figure 5:**
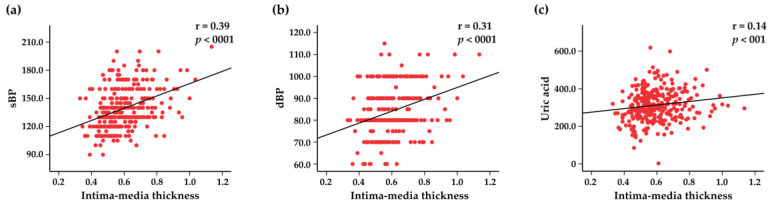
Scatter plots representing the correlations between intima–media thickness and systolic blood pressure (sBP) (**a**), diastolic blood pressure (dBP) (**b**), and blood uric acid levels (**c**).

Atherosclerotic plaques were identified in 76 (25%) subjects, with a mean age of 62.0 (95% CI: 60.3–63.7)–61.3 (95% CI: 58.8–63.7) in men and 62.6 (95% CI: 60.3–65.1) in women. The differences in subjects’ demographic, clinical, and laboratory characteristics based on the presence of carotid plaques are presented in Table 3. As can be observed, subjects with carotid plaques displayed higher values of systolic BP, diastolic BP, BMI, waist circumference, TC, LDL-C, uric acid, homocysteine, and hs-CRP (Table 3). The total number of plaques in ICA and CCA in men and women are represented in Table S2. The mean value of IMT of the right and the left CCA was 0.59 mm (95% CI: 0.5–0.6), and the mean total plaque area was 32.6 mm^2^ (95% CI: 26.6–38.5). The majority of subjects with plaques (97%) had stenosis less than 50%. Associations between the IMT and clinical variables are shown in Figure 5.

## 4. Discussion

Despite the advances in the treatment of stroke, prevention remains the most effective strategy, especially primary prevention, due to the fact that more than 76% of stroke cases are primary events. An international case-control study demonstrated that 90% of stroke cases are due to 10 risk factors: (1) arterial hypertension, (2) diabetes mellitus, (3) cardiac diseases, (4) current smoking, (5) abdominal obesity, (6) dyslipidemia, (7) physical inactivity, (8) alcohol consumption, (9) diet, and (10) psychosocial stress and depression [41]. Many of these modifiable stroke risk factors can be prevented and controlled by adopting a healthy lifestyle or seeking therapeutical interventions [26]. In our study we revealed that one of the most commonly encountered risk factors was abdominal obesity, which was shown to be an independent risk factor for stroke [42]. Moreover, several studies have evidenced that abdominal obesity, rather than BMI, is strongly associated with the risk of stroke [43]. In contrast, Meschia et al. demonstrated that only BMI was significantly associated with stroke in males, while waist–hip ratio was an independent predictor in women [44]. Considering age and sex, a study reported that excessive weight and abdominal adiposity were associated with the risk of stroke in older men compared to women [45]. Sex effects were also demonstrated in a study showing that the association between abdominal obesity and the risk of stroke was less pronounced in women compared to men [43]. Similar findings were reported by another study, which pointed out no differences between abdominal obesity and general obesity as predicting factors of stoke in women [46]. These data point to the necessity of adopting sex-specific approaches while studying the potential links between the measures of abdominal obesity and cerebrovascular risks. General obesity is another risk factor for different cardiovascular diseases [47] and is a largely preventable and treatable condition; however, the incidence of obesity has tripled in recent decades. Abdominal and general obesity were recognized as risk factors for high BP [48]. In our study population, both abdominal and general obesity were strongly associated with systolic and diastolic BP. General obesity was more prevalent in women than in men, while the prevalence of abdominal obesity was comparable between the sexes. We also observed that general and abdominal obesity increased with age, most frequently being encountered in the ≥50 years age group. General and abdominal obesity in this age group were identified in 61% and 85% of subjects, respectively, compared to 30% and 61% of subjects, respectively, in the 18–49 years age group.

The second risk factor by frequency identified in our study was high TC, which was observed more frequently in women than in men (61% vs. 39%, respectively). Different views exist regarding the role of TC as a risk factor for stroke. Thus, several studies showed that high TC is a risk factor for ischemic stroke [49,50], whereas other studies have shown a poor association between TC and stroke [51,52]. In a large population-based cohort study on 5,688,055 statin-naive subjects, high levels of TC were shown to be associated with a high risk of stroke even in young adults aged 20–39 years [53]. A meta-analysis on 1,022,276 individuals demonstrated that high TC was a strong risk factor for coronary heart disease, but had a small effect on the risk of stroke in both sexes [54]. Despite these controversies, TC in combination with other risk factors such as age, sex, smoking status, systolic BP, and HDL-C is a key component of cardiovascular risk prediction models widely applied in clinical settings. Blood homocysteine level is considered to be a modifiable risk factor for cardiovascular diseases, and even mild to moderate increases are associated with increased cardiovascular risk [51,52]. Elevated homocysteine levels are also associated with an increased risk of carotid atherosclerosis and stroke [44]. We identified high homocysteine values in 55% of subjects, of which 52% had atherosclerotic plaques. Moreover, we observed positive correlations between the homocysteine levels and systolic BP. Different studies showed the link between the homocysteine levels and BP, especially the systolic BP [55,56,57,58,59] and the risk of hypertension [59]. However, other studies reported that elevated homocysteine levels in hypertensive individuals are likely to be concomitant with rather than a precursor of hypertension [60]. In our study, hypertension was the fifth most frequently identified risk factor (in 44% of subjects); of these, 85% had signs of left ventricular hypertrophy, which is known to be a marker of and contributor to coronary events, stroke, heart failure, peripheral arterial disease, and cardiovascular mortality [61]. It is largely known that age is one of the most potent risk factors for stroke [44]. Our findings showed that higher age of the subjects was associated with higher values of both systolic and diastolic BP. Several previous studies demonstrated similar findings—a significant increase in systolic BP with age as well as a significant increase in diastolic BP, especially after the age of 60 years [62]. The prevalence of hypertension increases with advanced age [63,64] and is also sex-specific [64]. Inflammation, oxidative stress, and vascular dysfunction were proposed as putative mechanisms shared by both biological aging and hypertension development [65].

Increased IMT of CCA is associated with an increased risk of stroke [66]. The IMT can be considered an early common integrator of various traditional risk factors for stroke on the arterial wall [67]. We studied the relationship between IMT of CCA and other stroke risk factors, and found significant associations between IMT of CCA and systolic and diastolic BP, BMI, and waist circumference. It is also important to note that high levels of uric acid are strongly associated with carotid vascular disease and stroke [68]. Similarly, a strong correlation between the IMT and uric acid levels was observed in our study population. Several interesting findings emerged while contrasting the subjects with and without carotid plaques. Subjects with carotid plaques were older, more obese, and had higher values of systolic and diastolic BP. Additionally, factors such as TC, LDL-C, homocysteine, and hs-CRP, which are incriminated in the atherogenesis, were also elevated in the subpopulation of subjects with carotid plaques.

One particularly important finding of our study was the prevalence estimates of traditional risk factors in young adults. Our results revealed a relatively high prevalence of abdominal and general obesity as well as dyslipidemia, while hypertension was attested only in one-fourth of subjects in this age group. In young adults, ischemic stroke was thought to be related mainly to ‘rare’ risk factors and clinical entities that are very different from the ‘traditional’ vascular risk factors and etiologies observed in older patients with stroke [22]. However, population-based studies have evidenced that an increase in stroke incidence in young adults has been found to be associated with increasing prevalence of traditional vascular risk factors, such as hypertension, hypercholesterolemia, diabetes mellitus, and obesity [23,69,70]. Thus, hypertension is being identified in approximately 19–39% of all young adults with stroke, dyslipidemia in 17–60%, diabetes in 2–10%, and obesity in 10–20% of cases [22]. The prevalence of multiple traditional stroke risk factors among young adults with stroke has doubled over the decade from 2003–2004 through 2011–2012, as highlighted in recent reports [69]. The presented evidence suggests that prevention strategies targeting traditional risk factors are as important in young adults as in older individuals.

Abdominal obesity, dyslipidemia, hyperhomocysteinemia, general obesity, and arterial hypertension were the most prevalent risk factors identified in the Moldovan population. An alarming relatively high prevalence of these risk factors was observed in the younger age group, indicating the necessity of targeted preventive strategies for young adults to be undertaken on a national level in order to avert the development of cerebrovascular complications. Thereby, a focused, efficient, and comprehensive strategy for stroke prevention should be implemented by healthcare providers in the Republic of Moldova, and must include early detection and treatment of these conditions. An important aspect with any risk factor prevention strategy is that it should take into account the multiple risk factors that may be present in one individual and lead to cumulative risks for stroke [71,72], thus requiring multitargeted interventions. Based on the findings of our study, healthy individuals, patients, and physicians could better understand the impact of prevention strategies on the modification of identified risk factors that would reduce the burden on the country caused by stroke. By increasing the understanding of stroke risk factors and by improving national stroke prevention programs in our country, we will be able to prevent a significant number of strokes before they lead to death and disability. In this way, two major strategies [73,74] could be applied: the high-risk strategy and the mass (population-based) strategy. The high-risk one deals with individuals with a higher risk of developing stroke and includes lifestyle changes (i.e., reduced salt and alcohol intake, physical activity, weight loss, smoking cessation, etc.) and pharmacological interventions (i.e., BP and lipid lowering agents, antiplatelet medications, etc.). One significant drawback of this approach is that it neglects the individuals who are at low and moderate risk for stroke. The population-based strategy comprises widely implemented approaches to target the entire population, aiming to reduce the stroke risks. This strategy relies on national policy and legislative regulations and involves the use of mass media as well as health education in communities, schools, and workplaces to promote a healthy lifestyle. Nevertheless, a combined approach for stroke prevention comprising strategies for high-risk individuals and population-wide prevention strategies may be more cost-effective.

Several limitations apply to this study. First, the sample size is relatively small, which could weaken the study’s statistical power. The sample size required to conduct an epidemiological study substantially varies depending on the prevalence rate of the outcome measure and on the marginal errors [75]. Since hypertension and obesity reach or even exceed a prevalence rate of *p* = 30% (depending on the geographical region) [76,77], the sample size falls in a range between *n* = 143 and 896 included subjects [75]. Thus, our sample size of *n* = 300 could be sufficient to capture the prevalence of frequently encountered clinical outcome measures. Second, this study covered only one district area in the central region of the country, so it may not be able to represent other regions with potentially different characteristics. However, given the small geographical area of the Republic of Moldova (33,851 km^2^), which is inhabited by people sharing similar lifestyles throughout the country, comparable prevalence of risk factors might be assumed in the southern, central, and northern regions of the country. Third, history of hypertension, diabetes, and heart disease was obtained through subjects’ reports during the interviews without cross-validation with medical records, which might have under- or overestimated their prevalence. Since the subjects‘ assessment was conducted by an interdisciplinary team of trained physicians, the interviewer bias should be minimal. Fourth, some other risk factors such as patent foramen ovale and chronic autoimmune or infectious conditions could not be assessed. Nevertheless, their prevalence rate is low; hence, it is unlikely to essentially alter the distribution of identified risk factors in the studied population. Finally, since this study is an epidemiological study with a cross-sectional design, a certain degree of selection, participant, and prevalence bias are inevitably present.

Despite the limitations listed above, following strengths of the study are worthy of mention. First, our study provides, for the first time, a detailed description of stroke risk factors and their interrelations in the population of our country, thereby offering valuable evidence for preventive programs on a national level. Second, we showed that such traditional stroke risk factors as obesity, dyslipidemia, and hypertension are encountered relatively frequently in young adults, thus confirming the importance of evaluation of these risk factors in this age group. Third, we demonstrated distinct associations between various risk factors in apparently healthy subjects, in contrast to other studies that emphasized the associations between risk factors mainly in subjects already living with clinical conditions such as hypertension, diabetes or obesity. Fourth, one of the major strengths of our work is the analysis of blood biochemical abnormalities in patients with atherosclerotic plaques characterized by high homocysteine, uric acid, and hs-CRP levels, which may add to the existing evidence on pathophysiological links to carotid atherosclerotic disease.

## 5. Conclusions

The results of our study show a spectrum of various stroke risk factors, among which abdominal obesity, dyslipidemia, hyperhomocysteinemia, general obesity, and arterial hypertension were found to be the most frequent in the studied population. A relatively high frequency of these risk factors was also observed in younger adults. These findings indicate the presence of a high prevalence of modifiable risk factors and the necessity of elaboration of primary prevention strategies that will minimize the burden of stroke in the population of the Republic of Moldova.

## Figures and Tables

**Table 1:** Demographic, clinical, and prevalence characteristics of the study subjects.

	All Subjects, *n* = 300 (100%)	Women, *n* = 180 (60%)	Men, *n* = 120 (40 %)	*p*
**Age, mean ± SD**	49.91 ± 14.5	49.6 ± 13.8	50.3 ± 14.9	0.469
**Education, *n* (%)**				
School/college	204 (68.0)	123 (60.3)	81 (39.7)	0.932
University	96 (32.0)	57 (59.4)	39 (40.6)	
**Smoking, *n* (%)**				
Current smoker	31 (10.3)	3 (9.7)	28 (90.3)	**<0.001**
Former smoker	39 (13.0)	3 (7.7)	36 (92.3)	
Non-smoker	230 (76.7)	174 (75.7)	56 (24.3)	
**Alcohol consumption, *n* (%)**				
No consumption	129 (43)	100 (77.5)	29 (22.5)	**<0.001**
Moderate consumption <20–30 gr/day	143 (47.7)	73 (51.0)	70 (49.0)	
Excessive consumption >20–30 gr/day	28 (9.3)	7 (25.0)	21 (75.0)	
**Arterial hypertension, *n* (%)**				
Yes	131 (43.7)	83 (63.4)	48 (36.6)	0.513
No	159 (53.0)	92 (57.9)	67 (42.1)	
Unknown	10 (3.3)	5 (50.0)	5 (50.0)	
**Diabetes mellitus, *n* (%)**				
Yes	26 (8.7)	11 (42.3)	15 (57.7)	0.156
No	256 (85.3)	158 (61.7)	98 (38.3)	
Unknown	18 (6)	11 (61.1)	7 (38.9)	
**Atrial fibrillation, *n* (%)**				
Yes	25 (8.3)	13 (52.0)	12 (48.0)	0.546
No	192 (64.0)	114 (59.4)	78 (40.6)	
Unknown	83 (27.7)	53 (63.9)	30 (36.1)	
**Ischemic heart disease, *n* (%)**				
Yes	40 (13.3)	31 (77.5)	9 (22.5)	**0.026**
No	204 (68.0)	113 (55.4)	91 (44.6)	
Unknown	56 (18.7)	36 (64.3)	20 (35.7)	
**Family history of stroke, *n* (%)**				
Yes	90 (30.0)	57 (63.3)	33 (36.7)	0.742
No	198 (66.0)	116 (58.6)	82 (41.4)	
Unknown	12 (4.0)	7 (58.3)	5 (41.7)	
**Family history of acute myocardial infarction, *n* (%)**				
Yes	49 (16.3)	27(55.1)	22 (44.9)	0.63
No	219 (73.0)	135 (61.6)	84 (38.4)	
Unknown	32 (10.7)	18 (56.3)	14 (43.7)	
**BMI, mean ± SD, kg/m^2^**	30.1 ± 5.8	30.7 ± 6.2	29.2 ± 5.1	**0.022**
**BMI > 30 kg/m^2^, mean ± SD**	34.8 ± 4.1	35.6 ± 4.3	33.6 ± 3.4	**0.003**
**Abdominal circumference (cm)**	96.5 ± 14.4	94.8 ± 14.7	99.1 ± 13.7	**0.011**
**Abdominal obesity (≥80 cm—female; ≥94 cm—male), mean ± SD**	101.87 ± 11.7	99.2 ± 11.8	107.23 ± 9.41	**<0.001**

SD: standard deviation; BMI: body mass index.

**Table 2:** Distribution of risk factors in age groups.

	18–49 Years, *n* = 122 (41%)	>50 Years, *n* = 178 (59%)	*p*
Abdominal obesity, *n* (%)	74 (61)	152 (85)	**<0.001**
Dyslipidemia, *n* (%)	67 (55)	134 (75)	**<0.001**
Hyperhomocysteinemia, *n* (%)	58 (48)	108 (61)	**0.025**
General obesity, *n* (%)	37 (30)	108 (61)	**<0.001**
Hypertension, *n* (%)	21 (17)	110 (62)	**<0.001**
Family history of stroke, *n* (%)	42 (34)	48 (27)	0.07
Atherosclerotic plaques, *n* (%)	3 (3)	73 (41)	**<0.001**
Smoking, *n* (%)	14 (12)	17 (10)	0.55
Alcohol consumption, *n* (%)	7 (6)	21 (12)	0.16
Diabetes mellitus, *n* (%)	2 (2)	24 (13)	**<0.001**
Atrial fibrillation, *n* (%)	4 (3)	21 (12)	**<0.01**

**Table 3:** Subjects’ characteristics depend on the presence of carotid plaques (CP).

Characteristics	CP	Non-CP	*p*
**Total, *n* (%)**	76 (25.4)	224 (74.6)	NA
**Men, *n* (%)**	38 (50.0)	82 (36.6)	**0.043**
**Women, *n* (%)**	38 (50.0)	142 (63.4)	
**Age, mean** ± **SD**	61.9 ± 7.3	45.8 ± 13.7	**<0.001**
**Age group, *n* (%)**			
18–49 years	3 (3.9)	119 (53.1)	**<0.001**
≥50 years	73 (96.1)	105 (46.9)	
**Current smoker, *n* (%)**	12 (15.7)	19 ( 8.4)	0.06
**Diabetes, *n* (%)**	13 (17.1)	13 (5.8)	**0.002**
**sBP, mean ± SD, mmHg**	150.7 ± 21.5	135.2 ± 20.1	**<0.001**
**dBP, mean ± SD, mmHg**	90.4 ± 11.2	82.3 ± 90.3	**<0.001**
**BMI, mean ± SD, kg/m^2^**	31.4 ± 5.3	29.7 ± 5.9	**0.003**
**Waist circumference (cm)**	102.6 ± 13.0	94.4 ± 14.3	**<0.001**
**FBG, mean ± SD, mmol/L**	5.9 ± 1.8	5.3 ± 1.1	**<0.001**
**TC, mean ± SD, mmol/L**	5.6 ± 1.1	5.4 ± 1.1	**0.028**
**TG, mean ± SD, mmol/L**	1.4 ± 0.7	1.3 ± 0.7	0.071
**HDL-C, mean ± SD, mmol/L**	1.4 ± 0.4	1.5 ± 0.4	**<0.001**
**LDL-C, mean ± SD, mmol/L**	4.1 ± 1.0	3.7 ± 1.0	**<0.001**
**Uric acid, mean ± SD, μmol/L**	332.2 ± 93.4	306.2 ± 78.1	**0.018**
**Homocysteine, mean ± SD, mmol/L**	14.5 ± 4.1	12.8 ± 5.0	**<0.001**
**hs-CRP, mean ± SD**	4.2 ± 6.9	3.9 ± 15.2	**<0.001**

SD: standard deviation; sBP: systolic blood pressure; dBP: diastolic blood pressure; BMI: body mass index; FBG: fasting blood glucose; TC: total cholesterol; TG: triglycerides; HDL-C: high-density lipoprotein cholesterol; LDL-C: low-density lipoprotein cholesterol; hs-CRP: high-sensitivity C-reactive protein.

## Data Availability

The data that support the findings of this study are available from the corresponding author upon reasonable request.

## Supplementary Materials

The following supporting information can be downloaded at: https://www.mdpi.com/article/10.3390/jcdd10020035/s1, Table S1: ECG characteristics of the subjects; Table S2: Gender-wise distribution in number of plaques in internal carotid (ICA) and common carotid (CCA) arteries.
