# Supplementary material for: Dissecting the Spectrum of Stroke Risk Factors in an Apparently Healthy Population: Paving the Roadmap to Primary Stroke Prevention
Source: J Cardiovasc Dev Dis. 2023 Jan 20;10(2):35. doi: 10.3390/jcdd10020035 (PMC9965290; doi:10.3390/jcdd10020035)
Supplement: Supplementary file 1 [file jcdd-10-00035-s001.zip › jcdd-2095309-supplementary.pdf]

Supplementary material

Supplementary Table S1. ECG characteristics of the subjects

ECG characteristics	Total, n = 300	Women, n = 180	Men, n = 120	*P*
**Heart rate, mean ± SD**	72.6 ± 14.9	73.1 ± 12.5	71.8 ± 18.0	0.052
**LVH, n (%)**	159 (53.0)	104 (65.4)	55 (34.6)	**0.045**
**Atrial fibrillation, n (%)**	13 (4.3)	5 (38.5)	8 (61.5)	0.147

SD: standard deviation; LVH: left ventricular hypertrophy.

Supplementary Table S2. Gender-wise distribution in number of plaques in internal carotid (ICA) and common carotid (CCA) arteries.

Number of plaques, ICA and CCA	Men		Women	
	N	%	N	%
1	15	20	26	34
2	14	18	7	9
≥ 3	9	12	5	7
